# A case report of dengue hemorrhagic fever complicated with diabetic ketoacidosis in a child: challenges in clinical management

**DOI:** 10.1186/s12887-020-02300-9

**Published:** 2020-08-26

**Authors:** V. Thadchanamoorthy, Kavinda Dayasiri

**Affiliations:** 1 Faculty of Health Care Sciences, Eastern University, Batticaloa, Sri Lanka; 2 Base Hospital, Mahaoya, Sri Lanka

**Keywords:** DKA, Fluid therapy, DHF

## Abstract

**Background:**

Diabetic ketoacidosis (DKA) is a common presentation of type 1 diabetes mellitus (T1DM) precipitated by various bacterial and viral infections. Dengue infection is no exception for this and can be a precipitating factor for DKA. The presentation of DKA with dengue haemorrhagic fever (DHF) has been reported in adults. However, it is very rarely observed in children.

**Case presentation:**

We present the case of a paediatric patient who was previously healthy and subsequently, developed polyuria (above 3 ml/kg/hour), irritability and high blood glucose (724 mg/dl) during the critical phase of DHF. DKA was diagnosed with DHF and managed successfully with insulin and intravenous fluids. He recovered without complications and discharged home with follow up being arranged at the endocrinology clinic.

**Conclusions:**

When both DHF and DKA present together in a patient, meticulous monitoring of glycaemic control as well as fluid management is required to reduce the potential risk for severe complications of both conditions. Since there are no similar paediatric case reported in the literature, this case report might inspire paediatricians to anticipate the possibility of DKA in children with DHF.

## Background

Dengue has a wide spectrum of clinical manifestations which may be mild to severe and can be severe enough to cause death due to dengue shock syndrome. Worldwide estimates suggest that annual incidence of dengue fever and DHF has been 100 million and 500,000 respectively. Ninety percent of DHF cases are children under 15 years old [1, 2]. Dengue fever similar to other viral infections is known to precipitate diabetic ketoacidosis in patients with diabetes. Both insulin dependent and independent diabetes can increase the release of pro-inflammatory cytokines and intensify the risk of plasma leakage in dengue fever. Acute pancreatitis is a rare complication of severe dengue infection, which could be a contributory factor for diabetic ketoacidosis. The clear understanding of the comorbidity and mortality between the two diseases is vital in patient management during acute illness.

There is only limited research evidence with regard to actual fluid requirement during critical phase of dengue haemorrhagic fever as plasma leakage is dynamic and can occur at different rates across the critical phase [3]. Therefore, current practice of fluid management in DHF depends, to a greater extent, on expertise of the managing clinicians and a number of assumptions regarding evolution of plasma leakage. Urine output is considered as a reliable indicator of haemodynamic stability in patients with DHF and maintaining urine output between 0.5–1 ml/kg/hour is considered appropriate to prevent both shock and fluid overload that carry high risk of mortality. However, it is crucial that clinicians are mindful of potential confounding factors such as hyperglycemia. As a patient with dengue fever presents with hyperglycemia, urine output becomes an unreliable indicator of haemodynamic status and patient might have polyuria even during shock [4]. We report a child who was initially admitted for dengue fever and subsequently developed DHF associated with polyuria and irritability needing more fluids to maintain vital signs during the critical period of DHF. He was ultimately diagnosed as having type − 1 diabetes mellitus associated diabetic ketoacidosis with DHF. The report enlightens the importance of consideration of differential causes for surprisingly high urine output in patients with DHF associated shock and clinical decision making based on meticulous overall haemodynamic assessments. Management of this patient would be a thought-provoking and challenging task for clinicians and their teams.

## Case presentation

A 13-year-old previously healthy boy was admitted with fever, generalized body ache, headache, cough and mild diarrhea for 4 days and abdominal pain and vomiting for 2 days. His urine output was satisfactory. Dengue NSI antigen done on day 3 febrile illness was positive. He had no history of thirst, weight loss, and increased frequency of urination. On examination he was febrile (99.5F), ill looking, and flushed but was rational and heamodyanamically stable. Blood pressure had been 100/70 mmHg with pulse pressure of 30 mmHg. Pulse was of good volume and rate had been 155 / minute. His Complete Blood Count showed leukopenia (WBC- 1.5 × 10^3^/ cumm), and thrombocytopenia (platelet count - 100 × 10^3^/cumm). Haemoglobin was 13 g/dL, and haematocrit was 38. Random blood glucose on admission was 104 mg/dl. Abdominal examination showed 3 cm hepatomegaly and there was no clinical evidence of pleural effusion. He was provisionally diagnosed as having DHF and haemodynamic monitoring was commenced while he was on oral rehydration fluids at rate of 75 ml per hour. The child tolerated oral rehydration fluids well and did not need intravenous fluids including dextrose solutions.

On day five, he started to deteriorate with low volume pulse, tachycardia (rate of more than 180/min), cold clammy extremities and narrow pulse pressures whilst on intravenous 0.9% saline (4 ml/kg/hour) and oral fluids (1 ml/kg/hour). Clinical examination of lungs showed slight reduction of air entry on right side with vesicular breathing and no added sounds were heard. However, his urine output remained surprisingly satisfactory (more than1.5 mL/kg/hour). In addition, he became more irritable, thirsty, tachypnoic and had severe generalized abdominal tenderness whilst on two units of 0.9% saline 10 mL/kg boluses followed by 5 mL/kg/hour infusions. He continuously had disproportionately increased urine output (more than 2 mL/kg/hour) and pulse pressure varied between 15 to 20 mmHg. His Complete Blood Count showed WBC - 4.5 × 10^3^ (N^− 60^%, L-34%), haemoglobin - 16 g/dL, platelets - 60x10^3^mm/l, and haematocrit - 48. C-reactive protein (CRP) was elevated (12 mg/dl). Renal functions (Na- 140 mmol/L, K-4.3 mmol/L, serum creatinine 0.9 mg/dl) were normal apart from raised blood urea (60 mg/dL). Liver functions were deranged (Alanine transaminase − 240 IU/L, Aspartate transaminase-546 IU/L). Serum amylase was normal (44 U/L) Chest X-ray was normal apart from mild haziness all over the lungs. Ultrasound revealed mild ascites and bilateral pleural effusions. Capillary blood glucose was 724 mg/dl. He was transferred from local hospital to intensive care unit (ICU), in the tertiary care hospital for further management of diabetic ketoacidosis co-occurring with DHF. As he had high fever with unstable haemodynamic parameters and CRP was elevated, he was commenced broad spectrum empirical intravenous antibiotics. However, antibiotics were stopped following negative blood cultures.

In ICU, he had moderate to severe metabolic acidosis with arterial blood gas showing pH -7.17, pCO2-23 mmHg, pO2- 75 mmHg, HCO3- 12mmo/l and base excess-(− 14). Urine ketone bodies were positive. Blood ketones were not performed due to unavailability of this investigation in the hospital. He was resuscitated with dextran 40 with the dose of 10 ml/ kg once. Then he was started 0.9% saline with soluble insulin infusion at 0.1 u/kg/hour and blood glucose was monitored hourly until glucose levels dropped between 200 and 328 mg/dl. Intravenous fluid (0.9%saline) was adjusted between 5 and 7 ml/kg/h depending on the vital signs. We did not administer intravenous dextrose as it might have worsened hypovolaemia by the ongoing plasma leakage, producing more hydrostatic pressure and also producing osmotic diuresis. Instead child was advised to take foods which contained complex carbohydrate. Fluids were adjusted hourly according to pulse pressure which was more than minimum 20 mmHg and capillary refilling time was maintained below 2 s. The management was not guided both by urine output which had been more than expected and pulse rate due to presence of high fever. In addition, potassium was added to fluids as serum electrolyte revealed Na-140 mmol/L, and K-3.0 mmol/L while on insulin. As he improved after 24 h of critical period, his fluids and insulin were reduced to half. He was not commenced intravenous bicarbonate as repeat arterial blood gas showed improved findings (pH 7.32, PCO2-30 mmHg, PO2-80 mmHg, HCO2–18, Base excess (− 8)) following correction of dehydration and glucose with insulin. The lowest platelet count was 12x10^3^and renal function had been within normal range on the day 6 of illness. Intravenous Insulin was changed to subcutaneous insulin after 48 h of critical period and urine ketone bodies were noted to be negative.

After 72 h of ICU care, he was transferred to medical ward where he was continued on subcutaneously insulin and food according to dietician’s advice. Blood glucose, urine ketone body, renal functions, liver functions and hematological parameters were repeated until they were normalized. His HbA1C was 5.1% and Glutamic acid decarboxylase autoantibody had been positive (22 IU/L). Other type 1 diabetes related autoantibodies could not be performed due to limited financial resources in patient’s family. Both IgM and IgG dengue antibodies were positive on day 7 and dengue infection was notified to infection control team of the local hospital. Intravenous antibiotic was discontinued after 5 days with normal CRP. He was discharged after 2 weeks of hospital stay with the postprandial blood sugar being 146 mg/dl and fasting blood sugar being 100 mg/dl. Follow up was arranged at the endocrinology clinic. He was reviewed after 6 months and 1 year in the paediatric clinic and found to have been in good health and HbA1C was within normal range (5.3–5.5%). He is currently on insulin pump therapy under the care of paediatrician, paediatric endocrinologist and dietician. His growth and school performance had been within normal limits at one-year follow up.

## Discussion and conclusions

We report the case of this young boy who was initially admitted with dengue fever but subsequently developed DKA during the critical period of DHF. The rarity of this co-incidence in the paediatric age group and unexpected challenges in management of this child might inspire paediatricians. Altered haemostasis and plasma leakage have been two important pathophysiological mechanisms in DHF. Vascular leakage is caused by a transient increase in vascular permeability due to endothelial dysfunction and subsequent occurrence of haemoconcentration. The elevation of hematocrit greater than 20% is typically used as a cut-off to define the presence of leakage in dengue [5]. In patients with vascular leakage, excess intravenous fluid therapy can aggravate fluid accumulation and precipitates respiratory distress while substandard fluid treatment can cause shock. The osmotic diuresis in DKA results in large volume depletion. Typical total body water deficit among patients with DKA is 100 mL/kg of body weight, and the deficit becomes even higher with fluid loss in the phase of fluid leakage in DHF. Therefore, continuous monitoring and careful use of intravenous fluids are crucial in the management of patients with both DHF and DKA. The initial fluid therapy would be isotonic solutions to maintain satisfactory tissue perfusion and urine output should be at least 0.5 mL/kg/hour whilst insulin infusion is running. Intravenous fluids can be reduced gradually when plasma leakage decreases towards the end of the critical period which is usually signposted by an increase in urine output or decrease in hematocrit [6]. The reported child developed DKA during the critical period of DHF. We followed combined national and international guidelines for management of DKA and DHF. We managed the child with isotonic fluids and dextran 40 to restore the hypovolaemia. In addition, we were also guided by previous reports of dengue presenting with diabetes in adults [4, 7] as we could not find any reported cases in children. Literature reported in adults had been mainly dengue infection diagnosed in patients with insulin independent diabetes mellitus (type 2).

DHF was considered as a trigger factor for DKA in this patient who had previously undiagnosed diabetes mellitus. There were few case reports of dengue triggering diabetic ketoacidosis [4, 7, 8]. Supradish et al. reported the case of a 16-year-old Thai girl who presented in dengue shock showing signs of severe dehydration and ascites [7] and one other review article reported that those who had diabetes were two and half times as likely to have dengue hemorrhagic fever [9] The physiopathology of dengue hemorrhagic fever leads to amplification of the immune response following presence of heterotypic antibodies against a serotype of the dengue virus at the time of new infection [10]. Thus Type 1 diabetes mellitus is commonly associated with autoimmunity and the immune system may be persistently activated with signs of inflammation in tissues and capillaries, and is more likely to lead to inflammation and liberation of pro-inflammatory cytokines in tissues, particularly in the endothelium, explaining the higher risk of plasma leak in dengue fever [11].

Although ideal total body water deficit is 100 mL/kg in children with severe DKA, the actual deficit increases more than ideal deficit in the presence of fluid leakage. Both DHF related shock and fluid deficit due to DKA were managed in this patient with 0.9% saline with frequent monitoring of blood glucose, blood gases and haemodynamic parameters including pulse rate, and pulse pressure. Fluid intake was adjusted to keep urine output at least 0.5 mL/kg/hour and pulse pressure at least 20 mmHg. Early recognition of DKA and DHF are crucial in preventing complications related to both conditions.

The reported patient had hypovolaemia with surprisingly high urine output during the critical phase of DHF due to concurrent DKA. This situation in critical phase of DHF made fluid management more difficult even with central venous pressure monitoring. Fluids were adjusted every hour with insulin dose until the child reached recovery phase with meticulous blood glucose monitoring. As the reported child tolerated food well, cooperated with the management and was conscious throughout the critical period, he made a rapid and complete recovery of both complications without needing invasive monitoring. Meticulous non-invasive monitoring in the intensive care needed more human resources and added more mental stress to clinicians and their team who treated this child.

We further could not use any invasive monitoring to adjust the fluid management as his platelet had been low although invasive monitoring was available in the tertiary care hospital. This was another challenge the clinicians faced during fluid management as he had a high chance of internal bleeding while inserting the central cannulas. Fortunately, the child did not have bleeding at any time of illness and non-invasive monitoring sufficed. As this patient was admitted to local hospital where patient had been managed with limited facilities including human resources, timely diagnosis of coexisting diabetic mellitus was a challenge in the management of this child and possibly contributed for a delay in transfer to the intensive care unit. However, the diagnosis of diabetic ketoacidosis was made without much delay, and the child improved without developing any unacceptable complications. The authors recommend that urine output should be carefully reviewed in all patients with DHF on individual basis and differential causes for discordant urine output should be identified and managed without delay to prevent complications.

Dengue can rarely present with various atypical endocrinological manifestations in children. Every clinician must anticipate DKA in children with disproportionately high urine output during dengue infection even though it is rare in children. Scrupulous and frequent monitoring is an important step in identifying co-morbidities and treating these children. We were channeled with contradicting findings of urine output and vital parameters to make the diagnosis successfully.

## Data Availability

The data that support the findings of this case report are available from Medical Records Department, Batticaloa Teaching Hospital, but restrictions apply to the availability of these data, which were used under license for the current report and so are not publicly available. Data are, however, available from the authors upon reasonable request and with permission of Medical Records Department, Batticaloa Teaching Hospital, Sri Lanka.

## Abbreviations

DHF: Dengue haemorrhagic fever
DKA: Diabetic ketoacidosis
ALT: Alanine transaminase
AST: Aspartate transaminase
HbA1C: Haemoglobin A 1C
ICU: Intensive Care Unit
BU: Blood urea

## References

[1] Malavige GN, Fernando S, Fernando DJ, Seneviratne SL (2004). Review Dengue viral infections. Postgrad Med J.

[2] Kularatne SA (2015). Dengue fever. BMJ.

[3] Kularatne SA, Weerakoon KG, Munasinghe R, Ralapanawa UK, Pathirage M (2015). Trends of fluid requirement in dengue fever and dengue haemorrhagic fever: a single centre experience in Sri Lanka. BMC Res Notes.

[4] Dalugama C, Gawarammana IB (2017). Dengue hemorrhagic fever complicated with transient diabetic ketoacidosis: a case report. J Med Case Rep.

[5] Srikiatkhachorn A, Spiropoulou CF (2014). Vascular events in viral hemorrhagic fevers: a comparative study of dengue and hantaviruses. Cell Tissue Res.

[6] World Health Organization (2009). WHO Guidelines Approved by the Guidelines Review Committee. Dengue: Guidelines for Diagnosis, Treatment, Prevention and Control: New Edition.

[7] Supradish PO, Rienmanee N, Fuengfoo A, Kalayanarooj S (2011). Dengue hemorrhagic fever grade III with diabetic ketoacidosis: a case report. J Med Assoc Thail.

[8] Kitabchi AE, Umpierrez GE, Murphy MB, Kreisberg RA (2006). Hyperglycemic crises in adult patients with diabetes. A consensus statement from the American Diabetes Association. Diabetes Care.

[9] Figueiredo MA, Rodrigues LC, Barreto ML (2010). Allergies and diabetes as risk factors for dengue hemorrhagic fever: results of a case control study. PLoS Negl Trop Dis.

[10] Halstead SB (1981). The pathogenesis of dengue: molecular epidemiology in infections disease. Am J Epidemiol.

[11] Brown JM, Wilson TM, Metcalfe DD (2008). The mast cell and allergic diseases: role in pathogenesis and implications for therapy. Clin Exp Allergy.

